# Involvement of BcYak1 in the Regulation of Vegetative Differentiation and Adaptation to Oxidative Stress of *Botrytis cinerea*

**DOI:** 10.3389/fmicb.2018.00281

**Published:** 2018-02-21

**Authors:** Qianqian Yang, Jianan Zhang, Jicheng Hu, Xue Wang, Binna Lv, Wenxing Liang

**Affiliations:** The Key Laboratory of Integrated Crop Pest Management of Shandong Province, College of Plant Health and Medicine, Qingdao Agricultural University, Qingdao, China

**Keywords:** *Botrytis cinerea*, lysine acetylation, Yak1, vegetative differentiation, oxidative stress, virulence

## Abstract

Yak1, a member of the dual-specificity tyrosine phosphorylation-regulated protein kinases, plays an important role in diverse cellular processes in fungi. However, to date, the role of BcYak1 in *Botrytis cinerea*, the causal agent of gray mold diseases in various plant species, remains uncharacterized. Our previous study identified one lysine acetylation site (Lys252) in BcYak1, which is the first report of such a site in Yak1. In this study, the function of BcYak1 and its lysine acetylation site were investigated using gene disruption and site-directed mutagenesis. The gene deletion mutant ΔBcYak1 not only exhibits much lower pathogenicity, conidiation and sclerotium formation, but was also much more sensitive to H_2_O_2_ and the ergosterol biosynthesis inhibitor (EBI) triadimefon. The Lys252 site-directed mutagenesis mutant strain ΔBcYak1-K252Q (mimicking the acetylation of the site), however, only showed lower sclerotium formation and higher sensitivity to H_2_O_2_. These results indicate that *BcYAK1* is involved in the vegetative differentiation, adaptation to oxidative stress and triadimefon, and virulence of *B. cinerea*.

## Introduction

Lysine acetylation is one of the most common post-translational modifications (PTMs) to proteins in both eukaryotes and prokaryotes. Protein acetylation by reversible addition of an acetyl group to lysine residues could affect many important cellular and physiological processes, like cell-cycle regulation, cell morphology, metabolic pathways, protein-protein and protein-nucleic acid interactions, and enzymatic activity (Starai and Escalante-Semerena, 2004; Choudhary et al., 2009; Arif et al., 2010; Hou et al., 2010; Wang et al., 2010; Guan and Xiong, 2011; Nambi et al., 2013). Previous acetylome analysis in our laboratory identified 1582 lysine acetylation sites in 954 proteins in *Botrytis cinerea* that are involved in a variety of biological functions and localized to various cellular compartments (Lv et al., 2016). One of the acetylated proteins was Yak1 (XP_001555503.1), which was modified on the lysine residue, 252.

Yak1 was originally identified as a growth antagonist of the protein kinase A pathway in *Saccharomyces cerevisiae* and is a member of the dual-specificity tyrosine phosphorylation-regulated protein kinase family (Garrett and Broach, 1989; Becker and Joost, 1998). The Ser/Thr protein kinase activity of ScYak1 was altered by autophosphorylation of the second tyrosine residue in its YXY motif (Kassis et al., 2000). ScYak1 was phosphorylated by protein kinase A (PKA) *in vitro* and *in vivo* (Garrett et al., 1991; Zappacosta et al., 2002; Ptacek et al., 2005). Phosphorylation of ScYak1 by the catalytic PKA subunit Tpk1 could suppress the lethality associated with the loss of Tpk1 (Garrett and Broach, 1989; Zhu et al., 2000; Budovskaya et al., 2005). Further research showed that phosphorylation of Ser295 and Thr335 in ScYak1 plays an essential role in its localization and its binding of Bmh1 (a yeast 14-3-3 protein), respectively (Lee et al., 2011). The subcellular localization of ScYak1 could be altered. ScYak1 accumulates in the nucleus in response to glucose starvation or rapamycin-induced inhibition of the TOR pathway (Moriya et al., 2001; Wiatrowski and Carlson, 2003; Martin et al., 2004; Schmelzle et al., 2004). PKA-dependent phosphorylation of Ser295 and two minor sites of ScYak1 inhibits nuclear localization of this protein (Lee et al., 2011). ScYak1, along with Rim15 and Mck1, coordinates metabolic reprogramming to accumulate energy stores and activate anti-oxidant defense systems to ensure quiescence entry and lifespan extension in yeast (Cao et al., 2016).

Orthologs of Yak1 have been characterized in several fungi, including *Candida albicans, Candida glabrata, Penicillium marneffei, Trichoderma reesei, Fusarium graminearum, Aspergillus nidulans*, and *Magnaporthe oryzae*. Yak1 is indispensable for biofilm formation of *C. albicans* and *C. glabrata* (Iraqui et al., 2005; Goyard et al., 2008). In filamentous fungi, Yak1 is mainly involved in mycelium growth and stress response (Wang et al., 2011; Brown et al., 2013; De Souza et al., 2013; Suwunnakorn et al., 2014; Lv et al., 2015; Han et al., 2016). In *F. graminearum* and *M. oryzae*, deletion of Yak1 also affects conidiation and virulence (Wang et al., 2011; Han et al., 2016). Yak1 of *Arabidopsis thaliana* was identified as a dual-specificity protein kinase, and it plays an important role in ABA signaling and post-germination growth (Kim et al., 2015, 2016).

*B. cinerea*, the causing agent of gray mold which affects more than 400 plant species, can give rise to enormous financial impact because *B. cinerea* causes both pre- and post-harvest losses (Williamson et al., 2007; Dean et al., 2012). In order to determine the role of BcYak1 and its lysine acetylation in *B. cinerea*, we constructed and characterized *BcYAK1* mutants in this study. Deletion of *BcYAK1* not only led to reduced pathogenicity, lower conidiation and less sclerotium formation, but also increased sensitivity to H_2_O_2_ and the ergosterol biosynthesis inhibitor (EBI) triadimefon. Different from the deletion mutant, change of Lys252 to glutamine to mimic the acetylation status of this protein, resulted in only decreased sclerotium formation and increased sensitivity to H_2_O_2_. These results indicate that *BcYAK1* is involved in several processes in *B. cinerea*: vegetative differentiation, adaptation to oxidative stress and triadimefon, and virulence.

## Materials and methods

### Strains and culture conditions

Strain B05.10 of *B. cinerea* Pers.: Fr. [*B. fuckeliana* (de Bary) Whetzel] was an isolate from *Vitis vinifera* and was widely used as a standard reference strain (Quidde et al., 1999). *B. cinerea* was grown on potato dextrose agar (PDA, 200 g potato, 20 g dextrose, 20 g agar, and 1 L water) and minimal medium (MM, 10 mM K_2_HPO_4_, 10 mM KH_2_PO_4_, 4 mM (NH4)_2_SO_4_, 2.5 mM NaCl, 2 mM MgSO_4_, 0.45 mM CaCl_2_, 9 μM FeSO_4_, 10 mM glucose, and 1 L water, pH 6.9).

The amounts of conidium and sclerotium were counted after 10 days and 4 weeks incubation on PDA medium, respectively. Conidia of the strains were washed down from the plates, diluted to 5 ml with ddH_2_O, and then counted under a microscope. Growth tests under different stress conditions were performed on PDA plates supplemented with different agents including H_2_O_2_, triadimefon, NaCl, KCl, glycerol, sorbitol, Congo Red, SDS, iprodione as indicated (Yan et al., 2010). The percentage of mycelial radial growth inhibition (RGI) was calculated using the formula RGI = [(C–N)/(C−5)] × 100, where, C and N indicate colony diameter of the control and the treatment, respectively. Each experiment was repeated three times.

### Construction of *BcYAK1* deletion, complementation, and site-directed mutagenesis mutants

The gene deletion vector was constructed by inserting two flanking sequences of the *BcYAK1* gene into two sides of the *HPH* (hygromycin resistance) gene in the pBS-HPH1 vector (Dong et al., 2009). To construct the complementation vector, a NEO cassette containing a trpC promoter was amplified from plasmid pBS-RP-Red-A8-NEO (Dong et al., 2009) and cloned into the XhoI-HindIII sites of pBS to create plasmid pBS-neo. Then, a full-length *BcYAK1* gene including promoter and terminator regions was amplified from genomic DNA of the wild type strain B05.10 and cloned into NotI and SacI sites of pBS-neo to generate the complementation plasmid. The resulting gene deletion and complementation vectors were transformed into B05.10 and ΔBcYak1, respectively, to generate gene deletion and complementation mutants using protoplast formation and transformation of *B. cinerea* (Gronover et al., 2001; Jiang et al., 2011). Fusion PCR was employed to construct *B. cinerea* BcYak1-K252Q and BcYak1-K252R mutants (Yu et al., 2004). The primers used in this study were listed in the Supporting Information, Table S1. The mutants were verified by PCR and sequencing.

### Nucleic acid manipulations and qRT-PCR

Fungal genomic DNA was extracted as described previously (McDonald and Martinez, 1990). Plasmid DNA was isolated using plasmid miniprep purification kits (BioDev Co.; Beijing, China).

Expression levels of oxidative stress-related genes were measured by qRT-PCR. Mycelia of *B. cinerea* was cultured in potato dextrose broth (PDB) at for 2 days in a shaker and harvested after treating with 20 mM H_2_O_2_ for 2 h. RNA extraction was carried out using a protocol described previously (Yan et al., 2010). Reverse transcription was performed according to the manufacturer's instructions using Revert Aid H Minus First Strand cDNA Synthesis kits (Fermentas Life Sciences, Burlington, Canada). Ten micro liters cDNA were diluted to 50 μl with ddH_2_O and 1 μl diluted solution was used in each real time PCR assay. Real-time PCR amplifications were conducted in a CFX Connect ™ Real-Time System (Bio-Rad, Hercules, CA) using TAKARA SYBR Premix Ex Taq (TAKARA Bio Inc., Dalian, China) with the listed primers (Table S1).

PCR amplification with the primer pair β-tubulin-F and β-tubulin-R was performed for each sample to quantify the expression of the β-tubulin gene as a reference. Gene expression levels were calculated using the 2^−ΔΔCt^ method (Livak and Schmittgen, 2001). Three replicates were carried out for each sample.

### Pathogenicity and infection-related morphogenesis assays

Pathogenicity tests of *B. cinerea* were performed as previously described (Yang et al., 2013). Briefly, three-week-old tomato leaves were inoculated with 5 mm diameter plugs of 4-day-old cultures at 25°C with 16 h of daylight. After 3 days incubation, the lesion diameters were measured. The experiments were repeated three times. Infection-related morphogenesis was observed on onion epidermis using a published method (Doehlemann et al., 2006; Viaud et al., 2006).

### Western blot analysis

The *BcYAK1, BcYAK1-K252Q*, and *BcYAK1-K252R* genes were cloned into pYF11 plasmid by the yeast gap repair approach to generate the GFP fusion constructs BcYAK1-GFP, BcYAK1-K252Q-GFP, and BcYAK1-K252R-GFP (Bruno et al., 2004). Thereafter, the resulting fusion constructs were transformed into ΔBcYak1 after DNA sequencing verification to generate strains ΔBcYak1-GFP, ΔBcYak1-K252Q-GFP and ΔBcYak1-K252R-GFP, respectively. Protein extraction from the three strains were carried out as described (Gu et al., 2015). Then, GFP-BcYak1 was pulled down from soluble proteins of ΔBcYak1-GFP, ΔBcYak1-K252Q-GFP, and ΔBcYak1-K252R-GFP using anti-GFP antibody agarose beads (Beyotime, Shanghai, China) as described previously (Gu et al., 2015). In brief, 20 μl anti-GFP agarose beads were incubated overnight with 500 μg soluble proteins at 4°C. After three times washing, proteins were eluted from agarose. The elutants of the three strains were probed with pan anti-acetyllysine antibody (PTM Biolabs Inc., Hangzhou, China) and anti-GFP antibody (Beyotime, Shanghai, China) to detect the levels of BcYak1-GFP and its acetylation, respectively. Three biological replicates were performed for the Western blot analysis.

## Results

### Deletion and complementation of *BcYAK1* in *B. cinerea*

To investigate the role of *BcYAK1*, (Figure S1A) we generated single-gene deletion mutants of *BcYAK1* using a homologous recombination strategy. In total, 5 of 45 hygromycin-resistant transformants were identified as deletion mutants and they all showed identical phenotypic characteristics. To confirm that the phenotype of ΔBcYak1 is due to deletion of this gene, ΔBcYak1 was complemented with a full-length *BcYAK1* gene, resulting in a strain named ΔBcYak1-C.

### Involvement of *BcYAK1* in hyphal growth, conidium and sclerotium formation

**Figure 1:**
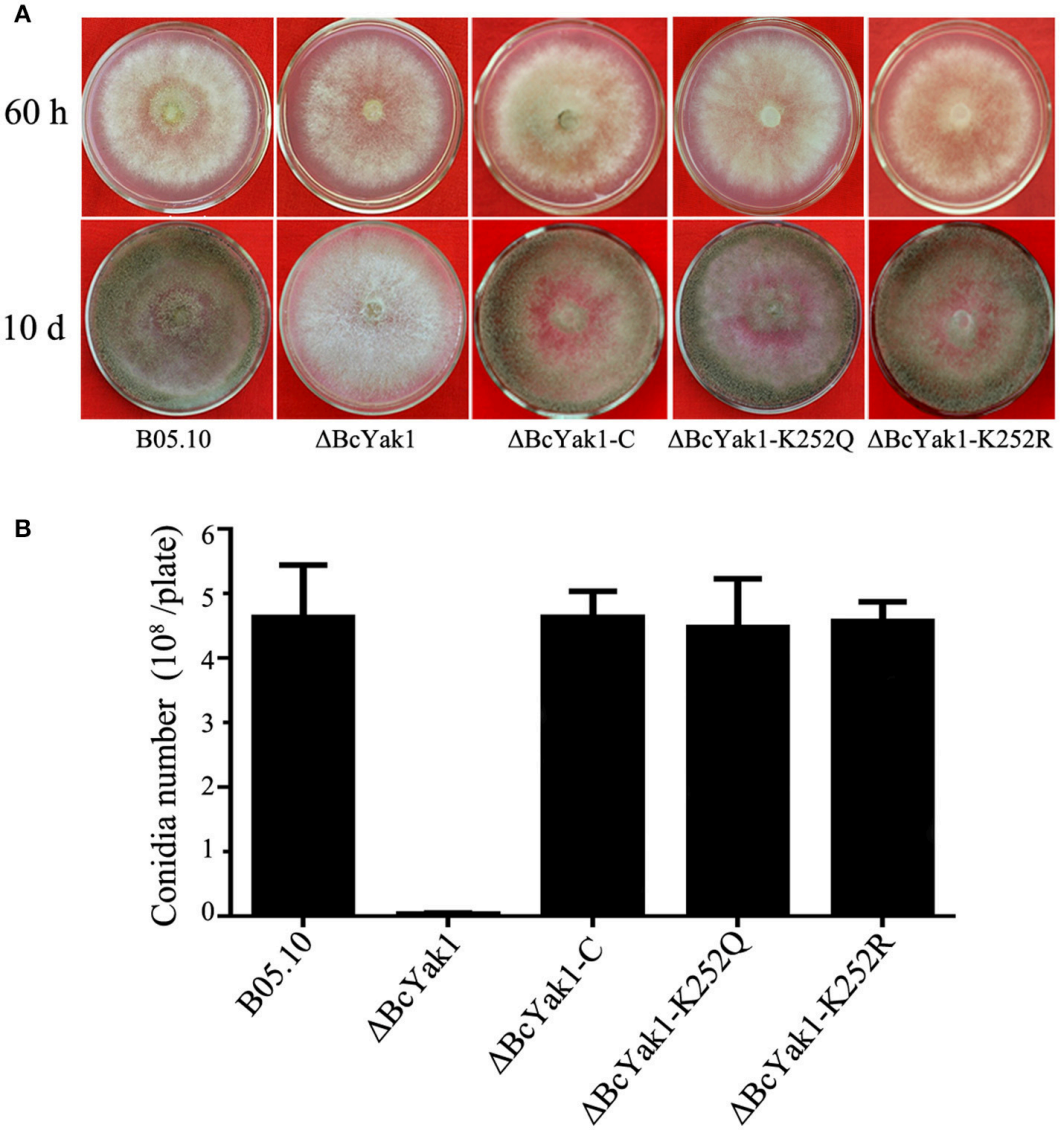
Impact of *BcYAK1* deletion and Lys252 site-directed mutagenesis on mycelial growth and conidium formation. **(A)** Mycelial growth of B05.10, ΔBcYak1, ΔBcYak1-C, ΔBcYak1-K252Q, and ΔBcYak1-K252R on PDA medium after 60 h and 10 days of incubation. **(B)** Number of conidia produced by each strain on PDA plates (diameter = 6 cm). Bars denote standard errors from three replications.

**Figure 2:**
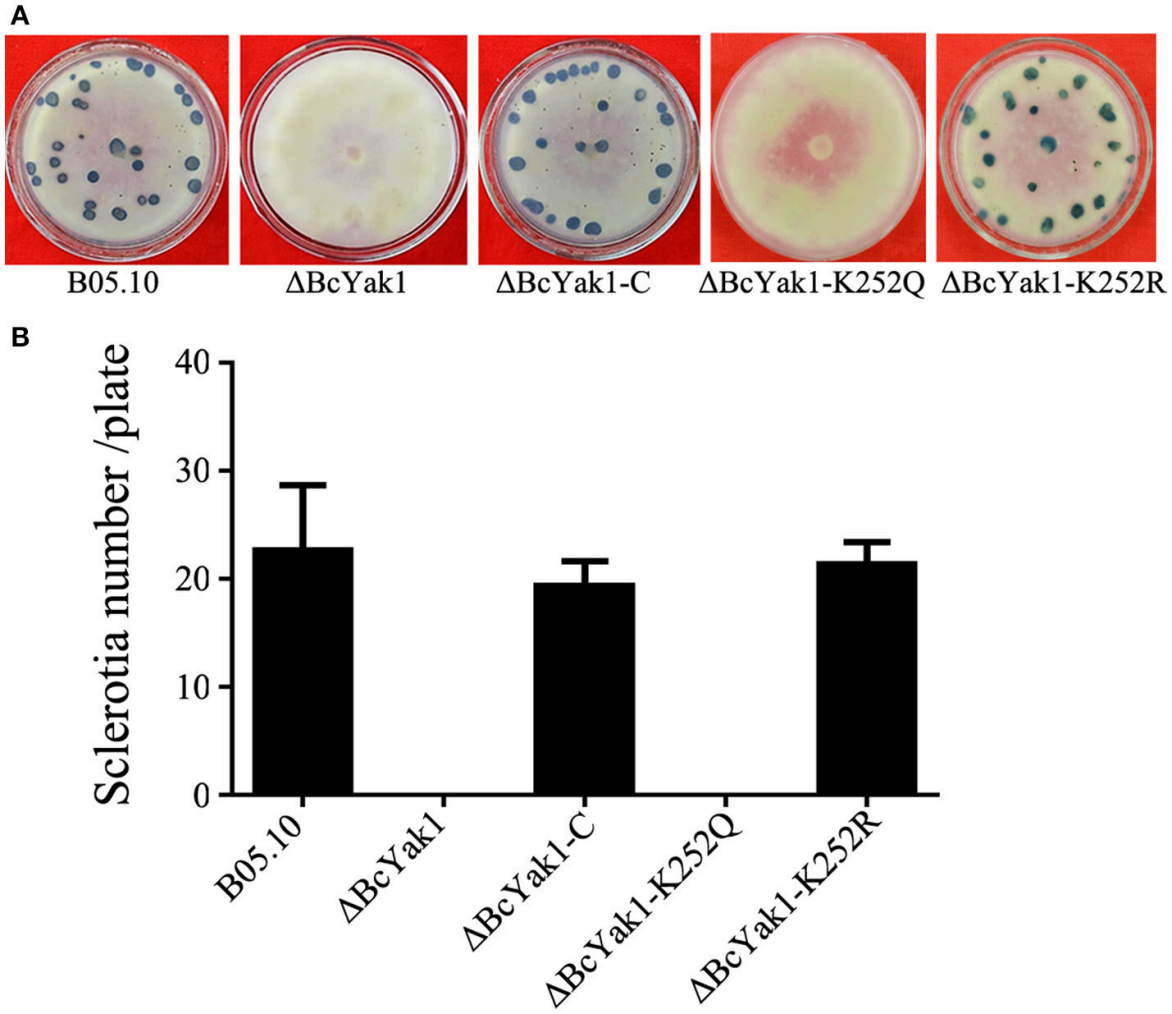
Impact of *BcYAK1* deletion and Lys252 site-directed mutagenesis on sclerotium formation. **(A)** Sclerotium formation of B05.10, ΔBcYak1, ΔBcYak1-C, ΔBcYak1-K252Q, and ΔBcYak1-K252R. The strains were incubated on PDA plates at for 4 weeks in darkness. **(B)** The number of sclerotia produced by each strain. Bars denote standard errors from three experiments.

The mycelial growth rate and the surface hydrophobicity of ΔBcYak1 was similar to that of the wild-type parent B05.10 (Figure 1A, Figure S2). However, after incubating on PDA for 10 days, ΔBcYak1 produced far fewer conidia than the wild-type parent and the complemented transformant (Figure 1B). Since sclerotial formation is an important survival mechanism of *B. cinerea* in nature (Williamson et al., 2007), the role of *BcYAK1* in sclerotial formation was investigated. After 4 weeks of incubation in the dark, ΔBcYak1 produced significantly fewer sclerotia than B05.10 and the complemented transformant ΔBcYak1-C (Figure 2). These results indicated that BcYak1 plays a role in the conidium and sclerotium formation of *B. cinerea*.

### Requirement of BcYak1 for full pathogenicity of *B. cinerea*

**Figure 3:**
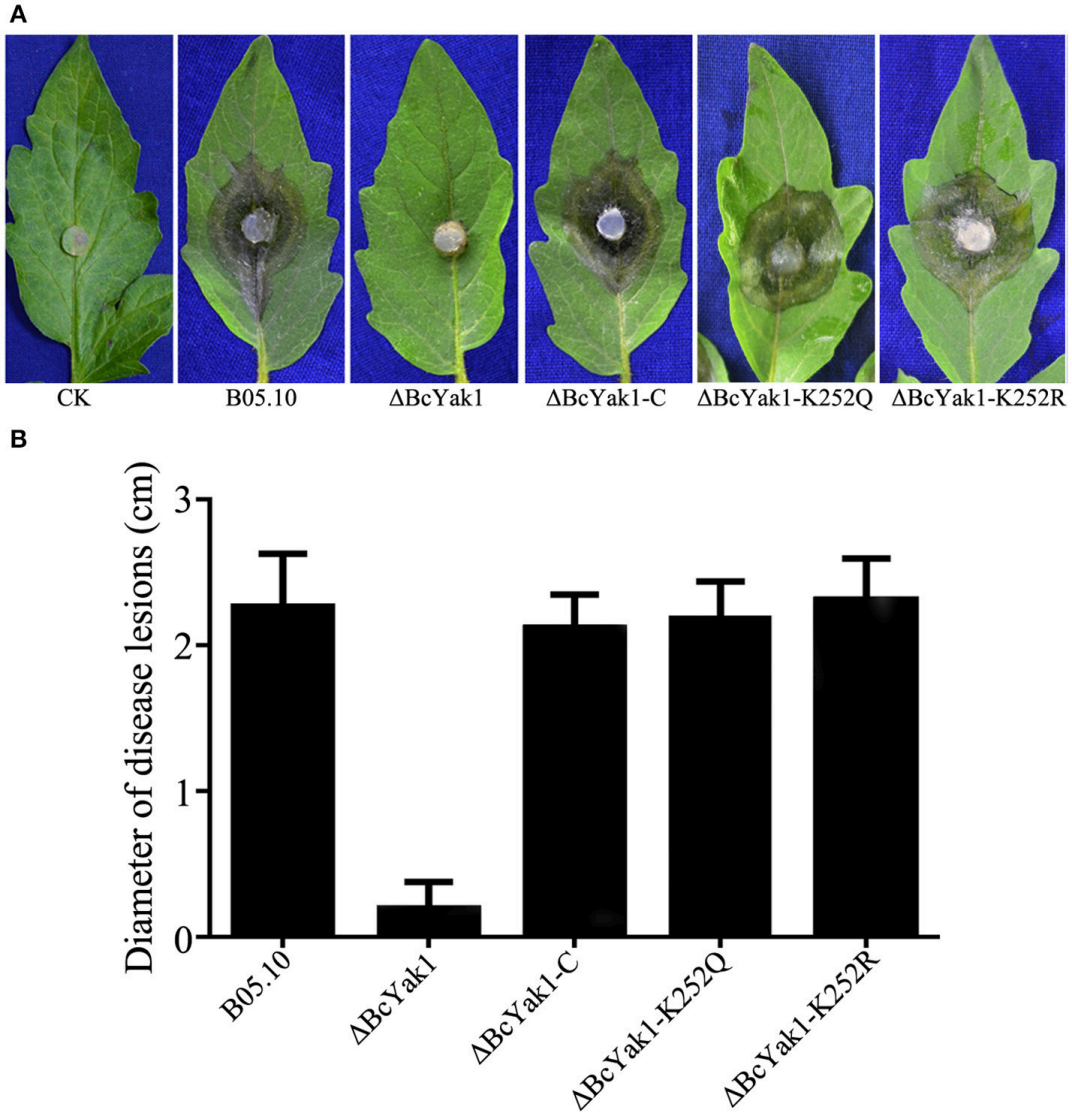
Requirement of BcYak1 for full pathogenicity of *B. cinerea*. Pathogenicity assays on tomato leaves following inoculation with B05.10, ΔBcYak1, ΔBcYak1-C, ΔBcYak1-K252Q, ΔBcYak1-K252R, and a negative control (CK). **(A)** Disease symptoms caused by each strain on wounded tomato leaves. The pictures were taken after 3 days inoculation. **(B)** Diameter of disease lesions on tomato leaves after 3 days inoculation. Bars denote standard errors of three replications.

**Figure 4:**
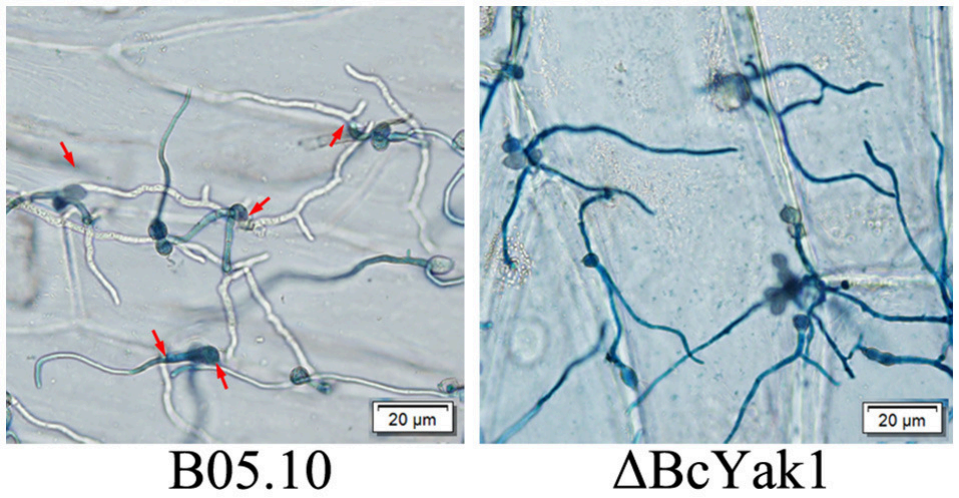
Onion epidermis penetration by B05.10 and ΔBcYak1. Pictures were taken after 14 hours inoculation of onion epidermis with conidia of B05.10 and ΔBcYak1. Infection sites were indicated by red arrows and hyphae growing within the plant cells (not stained) were observed clearly.

To test the role of *BcYAK1* in pathogenicity, we performed an infection test on tomato leaves. As shown in Figure 3, ΔBcYak1 showed reduced infection in the assay. Three days after inoculation, the mutant caused primary lesions on tomato leaves, while spreading lesions were formed by B05.10 and the complemented mutant ΔBcYak1-C (Figure 3). To analyze this pathogenicity defect of ΔBcYak1 in detail, we performed onion penetration assays. Compared with wild-type, the ΔBcYak1 germlings were unable to penetrate onion epidermis cells after 14 h of incubation, indicating that BcYak1 affected the penetration efficiency of *B. cinerea* (Figure 4).

### Effects of *BcYAK1* deletion on the sensitivity of *B. cinerea* to stresses

**Figure 5:**
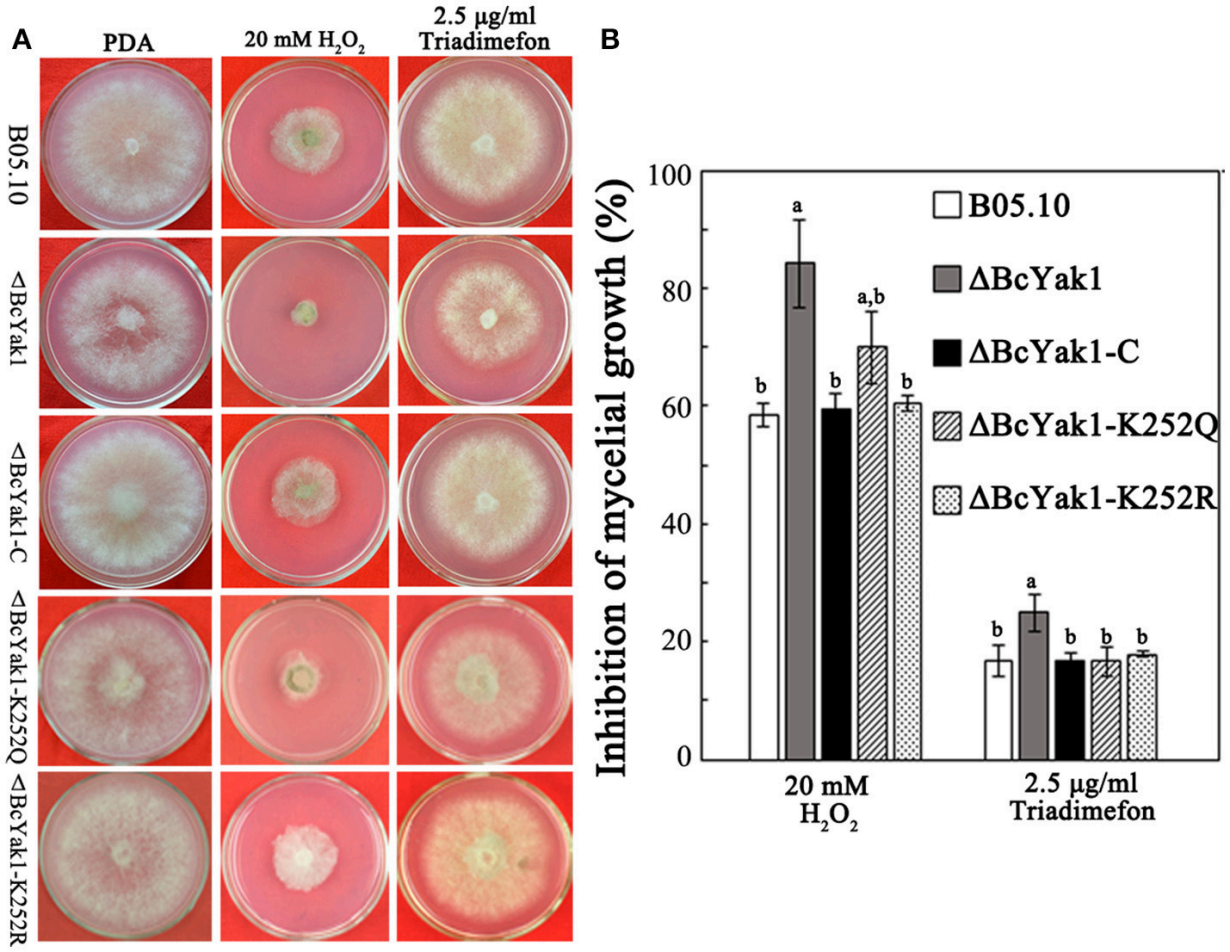
Sensitivity of B05.10, ΔBcYak1, ΔBcYak1-C, ΔBcYak1-K252Q and ΔBcYak1-K252R to H_2_O_2_ and triadimefon. **(A)** Sensitivity of the strains grown on PDA amended with H_2_O_2_ and triadimefon at the concentrations indicated in the figure. The pictures were taken after 2 days incubation at 25°C. **(B)** Inhibition of mycelial growth compared with non-treatment. Bars denote standard errors from three experiments. Statistical tests were carried out using Tukey test for multiple comparisons and values on the bars followed by the same letter are not significantly different at *P* = 0.05.

We also investigated the sensitivity of the mutants to oxidative and other stresses because oxidative tolerance levels affect the virulence of *B. cinerea*. Figure 5 shows that ΔBcYak1, compared to B05.10 and the complemented transformant ΔBcYak1-C, exhibited increased sensitivity to H_2_O_2_. In addition, ΔBcYak1 was sensitive to triadimefon, which inhibits fungal ergosterol biosynthesis (Yan et al., 2011), but not to osmotic stress (NaCl, KCl, sorbitol, and glycerol), cell wall stress (congo red), iprodione and other carbon sources (glucose and sucrose; Figure S3).

### Functional analysis of BcYak1 acetylation on lysine 252

**Figure 6:**
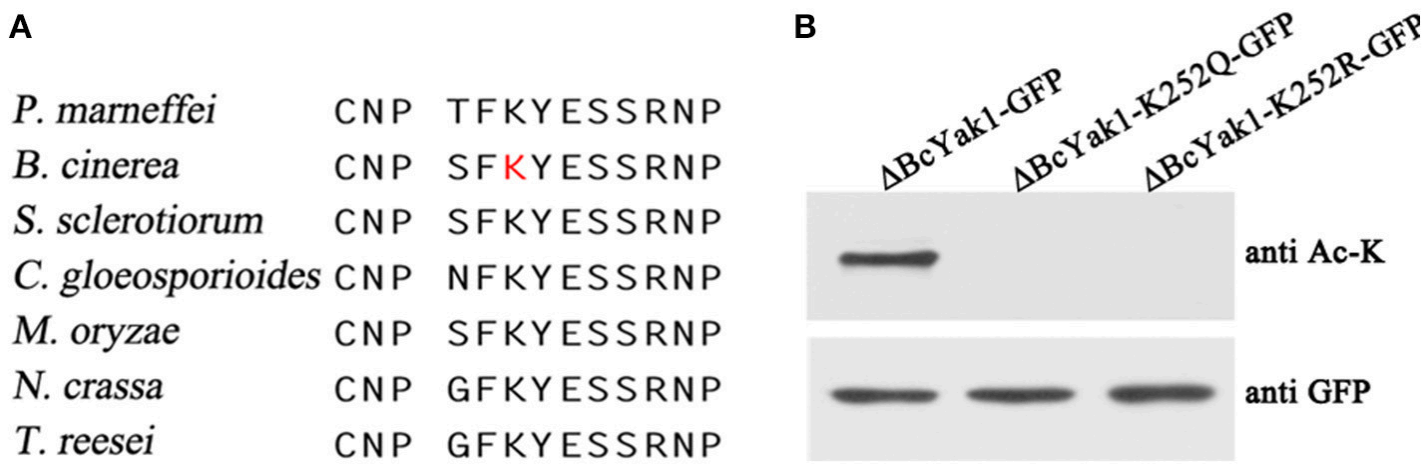
BcYak1 is acetylated at lysine 252. **(A)** The Lys252 site in Yak1 is relatively conserved. The amino acid sequences bracketing Lys252 in proteins from different species were aligned. The lysine acetylation site of BcYak1 is shown as a red letter. **(B)** Acetylation and protein level of wild-type and mutant BcYak1. BcYak1-GFP, BcYak1-K252Q-GFP, and BcYak1-K252R-GFP were expressed in ΔBcYak1. GFP-BcYak1 was pulled down from extracts of the mycelium using anti-GFP antibody agarose beads. The acetylation levels of BcYak1 were determined by anti-acetyllysine antibody, and the amount of protein was calculated using anti-GFP antibody.

BcYak1 was identified as a putative substrate of acetylation in previous proteomics studies (Lv et al., 2016). The acetylation site, Lys252, is conserved in Yak1-like proteins in several fungi (Figure 6A). To confirm acetylation at this site, we mutated lysine 252 to glutamine (Q) and arginine (R), respectively, and determined their acetylation level using a pan anti-acetyllysine antibody. Glutamine and arginine mimic acetylated and unacetylated lysine, respectively (Schwer et al., 2006; Li et al., 2007). As shown in Figure 6B, no acetylation was detected in the ΔBcYak1-K252Q-GFP and ΔBcYak1-K252R-GFP mutants, indicating that Lys252 is the only acetylation site in this protein.

Phenotypes of the two mutants were analyzed. ΔBcYak1-K252Q and ΔBcYak1-K252R produced similar amounts of conidia as B05.10 (Figures 1, 2), and they both were not defective in virulence (Figure 3). However, like ΔBcYak1, ΔBcYak1-K252Q, but not ΔBcYak1-K252R, produced significantly fewer sclerotia than B05.10. In addition, ΔBcYak1-K252Q, but not ΔBcYak1-K252R, was more sensitive to H_2_O_2_ than B05.10, but not as sensitive as ΔBcYak1 (Figure 5). Although deletion of BcYak1 led to increased sensitivity to triadimefon, the sensitivity of ΔBcYak1-K252Q and ΔBcYak1-K252R to this chemical was the same as that of wild type. Consistent with the behavior of ΔBcYak1, the sensitivity of ΔBcYak1-K252Q and ΔBcYak1-K252R to osmotic stress (NaCl, KCl, sorbitol and glycerol), cell wall stress (congo red), iprodione and different carbon source (glucose and sucrose) was similar to B05.10 (Figure S3). Based on these results, we conclude that Lys252 acetylation in BcYak1 affects oxidative stress sensitivity and sclerotia formation in *B. cinerea*.

**Figure 7:**
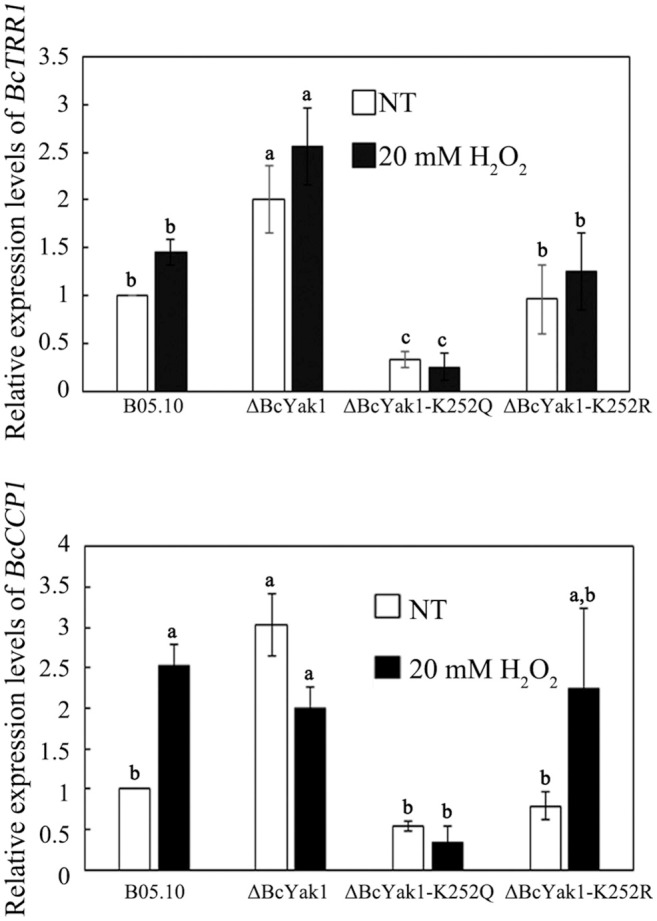
Relative expression level of *BcTRR1* and *BcCCP1* in B05.10, ΔBcYak1, and ΔBcYak1-K252Q. RNA samples were extracted from mycelia grown in PDB for 2 days following treatment with 20 mM H_2_O_2_ for 2 h. The culture without treatment was used as the control (NT). Bars denote standard errors from three experiments. Statistical tests were carried out using Tukey test for multiple comparisons and values on the bars followed by the same letter are not significantly different at *P* = 0.05.

To further investigate the oxidative stress sensitivity of ΔBcYak1 and ΔBcYak1-K252Q, we measured the expression levels of two oxidative stress response genes: *BcTRR1* and *BcCCP1*. In *S. cerevisiae, TRR1* (encoding mitochondrial cytochrome *c* peroxidase) and *CCP1* (encoding thioredoxin reductase) are both Yap1p and Skn7p-dependent oxidative stress response genes (Charizanis et al., 1999; He and Fassler, 2010; Morgan et al., 2014). Figure 7 shows that both *BcTRR1* and *BcCCP1* were up-regulated in response to H_2_O_2_ in the wild type and the ΔBcYak1-K252R strains. Interestingly, although deletion of BcYak1 caused this strain, compared to B05.10, to express more *BcTRR1*, the expression level of *BcCCP1* was much reduced after H_2_O_2_ treatment in this strain. Different from these three strains, ΔBcYak1-K252Q expressed relatively lower levels of *BcTRR1* and *BcCCP1*. These result further confirmed that Lys252 acetylation of BcYak1 played an important role in the response of *B. cinerea* to oxidative stress.

## Discussion

In this study, we investigated the functional roles of the *BcYAK1* gene in *B. cinerea* and found that BcYak1 has one lysine acetylation site, Lys252. We first disrupted the gene and characterized this mutant, ΔBcYak1, which exhibits severe defects in conidium and sclerotium formation (Figure 1, 2). These results are in agreement with experiments on Yak1 from *F. graminearum*, Moyak1 from *M. oryzae* and Tryak1 from *T. reesei* (Wang et al., 2011; Lv et al., 2015; Han et al., 2016). We thus speculate that Yak1 might be involved in the regulation of conidium formation-related genes in these fungi. However, the regulation mechanisms involving Yak1 remain poorly understood, and further research such as a comparative analysis of transcription profiles might provide more information. While the surface hydrophobicity of ΔMoyak1 was lost due to dramatically changed expression of hydrophobin-coding genes, the surface hydrophobicity of ΔBcYak1 was intact (Figure S2).

BcYak1 was also an important virulence determinant, and the involvement of Yak1 in virulence has been reported in two other fungal species: *F. graminearum* and *M. oryzae* (Wang et al., 2011; Han et al., 2016). ΔMoyak1 was unable to develop appressoria on an inductive surface, but it formed appressoria of abnormal morphology in response to exogenous cyclic adenosine-5-monophosphate and host-driven signals; these appressoria were all defective in penetrating host tissues due to abnormalities in glycogen and lipid metabolism, turgor generation and cell wall integrity (Han et al., 2016). Deletion of *BcYAK1* also compromised the penetration ability of *B. cinerea*, indicating that the reduced virulence of the *BcYAK1* mutant was likely due, as least in part, to defects in the penetration of host cells (Figure 4).

Although Yak1 in yeast and *T. reesei* are involved in the regulation of carbon source-sensing and carbon source-induced signals (Wiatrowski and Carlson, 2003; Lv et al., 2015), the hyphal extension rate of ΔBcYak1 on glucose and sucrose was similar to that of the parental strain (Figure S3). These results indicated that BcYak1 might not be involved in the glucose starvation response of *B. cinerea*. Growth on other carbon sources needs further investigation. Yak1 contributes to the adaptation of yeast cells to stress conditions other than glucose starvation stress. The involvement of Yak1 orthologs in stress response, similar to that of *S. cerevisiae* Yak1, has also been reported in *P. marneffei, T. reesei* and *F. graminearum* (Wang et al., 2011; Suwunnakorn et al., 2014; Lv et al., 2015). The deletion of Tryak1 in *T. reesei* led to increased sensitivity to osmotic (NaCl), oxidative (H_2_O_2_) and cell wall damage (calcofluor white and congo red) stresses (Lv et al., 2015). ΔBcYak1 exhibited increased sensitivity to H_2_O_2_, but not to osmotic stresses, congo red, SDS and iprodione (Figure S3). ΔBcYak1 also became more sensitive to the EBI triadimefon (Figure 5). These phenotypes are in common with two response regulator proteins of the high osmolarity glycerol (HOG) signaling pathway in *B. cinerea*, BRrg1 and BcSkn7, whose deletion also resulted in increased sensitivity to oxidative stresses and EBIs (Yan et al., 2011; Yang et al., 2015). HOG signaling pathway plays an important role in the response of fungi to various environmental stresses, including osmotic, oxidative and fungicide (iprodione) stresses. However, the molecular mechanisms by which BRrg1 and BcSkn7 are involved in the regulation of ergosterol biosynthesis in *B. cinerea* remain unclear. Since *skn7* and the downstream component of *rrg1, hog1*, were responsible for the regulation of the expression of many genes under stresses in *S. cerevisiae*, BcYak1 may share the regulation of certain genes. Investigating the targets of BcYak1 would thus be very interesting.

Lysine acetylation is one of the most common PTMs to proteins. Nε-lysine acetylation can change protein conformations and/or charges, thus altering DNA-binding affinity, enzymatic activity, protein stability, sub-cellular localization, and protein-protein interactions (Yang and Seto, 2008). Our previous study showed that BcYak1 contains one lysine acetylation site at Lys252 (Lv et al., 2016). Site-directed mutagenesis in this study confirmed that Lys252 is the only acetylation site in BcYak1. While ΔBcYak1-K252R showed phenotypes similar to that of B05.10, mutation of Lys252 to glutamine led to decreased sclerotial formation and increased sensitivity to H_2_O_2_ (Figure 5). Further study showed that the expression of two genes that respond to oxidative stress, including *TRR1* and *CCP1*, were significantly down-regulated in ΔBcYak1-K252Q mutant (Figure 7). These results indicate that acetylation of BcYak1 plays a role in the regulation of oxidative stress response genes. It is reasonable to hypothesize that acetylation does not greatly affect the function of BcYak1 considering that Lys252 is not distributed in the conserved domain PKc_YAK1 (Figure S1B). One possibility is that Yak1 represses the expression of both *TRR1* and *CCP1*, and this repression is released under oxidative stress which probably requires deacetylation of this protein. Additional studies will be needed to further clarify this regulatory mechanism.

## Conclusion

In summary, the Yak1 protein of *B. cinerea* plays an important role in pathogenicity, conidiation and sclerotium formation, and response to H_2_O_2_ and triadimefon. Acetylation of BcYak1 on the lysine residue, 252, affects sclerotium formation and H_2_O_2_ sensitivity of *B. cinerea*.

## Author contributions

QY and WL: Generated hypothesis and planned experiments; QY, JZ, JH, XW, and BL: Performed experiments; QY and WL: Wrote the paper; All other authors provided comments on the manuscript.

### Conflict of interest statement

The authors declare that the research was conducted in the absence of any commercial or financial relationships that could be construed as a potential conflict of interest.

## Supplementary material

Figure S1 Phylogenetic analysis and alignments of Yak1 from *B. cinerea* and other fungal species. **(A)** Phylogenetic analysis of amino acid sequence of Yak1 from *A. nidulans* (XP_664708), *A. thaliana* (NP_001031970.1), *Aspergillus fumigatus* (EDP47316.1), *B. cinerea* (XP_001555503.1), *C. albicans* (ACA23214.1), *C. gloeosporioides* (EQB46747), *Cryptococcus neoformans* (XP_572873.1), *Drosophila melanogaster* (CAA50065.1), *F. graminearum* (ESU11377), *M. oryzae* (XP_003717171.1), *Neurospora crassa* (EAA33897.2), *P. marneffei* (XP_002148404.1), *Phytophthora infestans* (XP_002898528.1), *S. cerevisiae* (CAA89437.1), *Sclerotinia sclerotiorum* (XP_001598012.1), and *T. reesei* (XP_006961091). The displayed tree was obtained by a Multiple Alignment method using DNAMAN software. **(B)** BcYak1 containing one conserved domain, PKc_YAK1, which was identified by SMART (http://smart.embl-heidelberg.de/). The lysine acetylation site of BcYak1 is indicated by the arrow.

Figure S2 The hydrophobicity of the surface mycelia of B05.10, ΔBcYak1, ΔBcYak1-C, ΔBcYak1-K252Q and ΔBcYak1-K252R. On each of the fungal colonies, 3 drops (15 μl each) of solution containing 0.2% SDS and 2.5% bromophenol blue were pipetted on the colony surface and photographed 10 min later.

Figure S3 Sensitivity of B05.10, ΔBcYak1, ΔBcYak1-C, ΔBcYak1-K252Q, and ΔBcYak1-K252R to stresses. Inhibition of mycelial growth among all the strains after 3 days incubation on PDA amended with each compound, as indicated in the figure. Bars denote standard errors from three experiments.

Table S1 Primers used in the study.
