# Supplementary figures and images for: Involvement of BcYak1 in the Regulation of Vegetative Differentiation and Adaptation to Oxidative Stress of Botrytis cinerea
Source: Front Microbiol. 2018 Feb 21;9:281. doi: 10.3389/fmicb.2018.00281 (PMC5826331; doi:10.3389/fmicb.2018.00281)

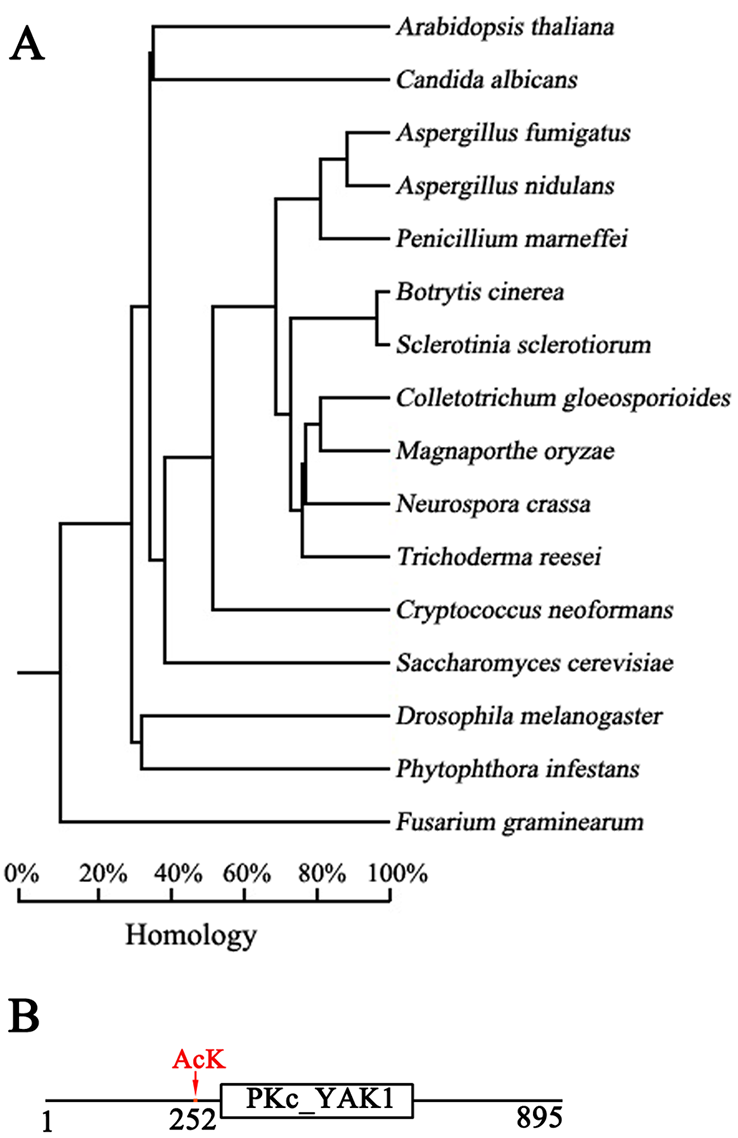

Supplement: Figure S1 — Phylogenetic analysis and alignments of Yak1 from B. cinerea and other fungal species. (A) Phylogenetic analysis of amino acid sequence of Yak1 from A. nidulans (XP_664708), A. thaliana (NP_001031970.1), Aspergillus fumigatus (EDP47316.1), B. cinerea (XP_001555503.1), C. albicans (ACA23214.1), C. gloeosporioides (EQB46747), Cryptococcus neoformans (XP_572873.1), Drosophila melanogaster (CAA50065.1), F. graminearum (ESU11377), M. oryzae (XP_003717171.1), Neurospora crassa (EAA33897.2), P. marneffei (XP_002148404.1), Phytophthora infestans (XP_002898528.1), S. cerevisiae (CAA89437.1), Sclerotinia sclerotiorum (XP_001598012.1), and T. reesei (XP_006961091). The displayed tree was obtained by a Multiple Alignment method using DNAMAN software. (B) BcYak1 containing one conserved domain, PKc_YAK1, which was identified by SMART (http://smart.embl-heidelberg.de/). The lysine acetylation site of BcYak1 is indicated by the arrow. [file Image1.TIF]

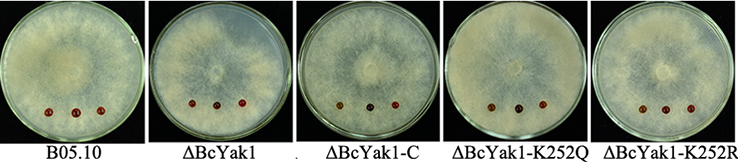

Supplement: Figure S2 — The hydrophobicity of the surface mycelia of B05.10, ΔBcYak1, ΔBcYak1-C, ΔBcYak1-K252Q and ΔBcYak1-K252R. On each of the fungal colonies, 3 drops (15 μl each) of solution containing 0.2% SDS and 2.5% bromophenol blue were pipetted on the colony surface and photographed 10 min later. [file Image2.TIF]

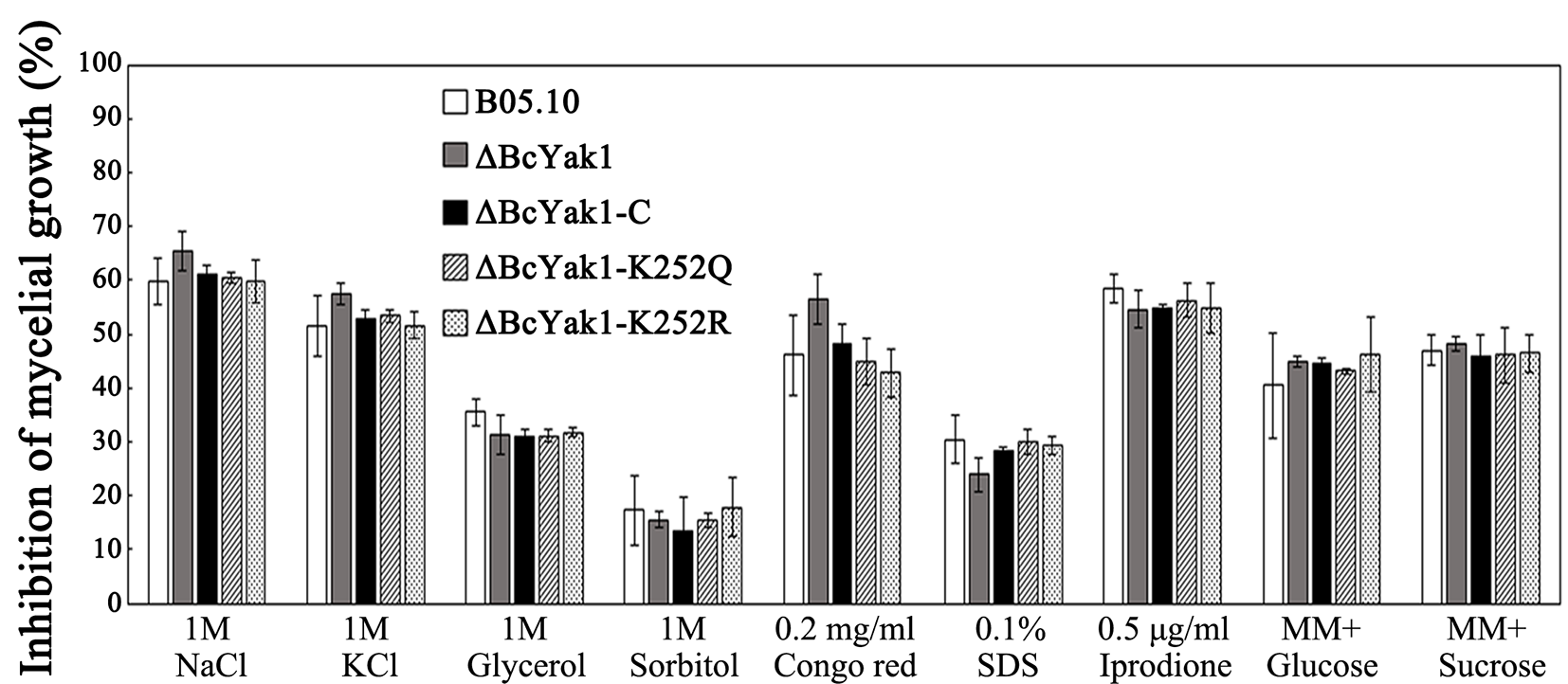

Supplement: Figure S3 — Sensitivity of B05.10, ΔBcYak1, ΔBcYak1-C, ΔBcYak1-K252Q, and ΔBcYak1-K252R to stresses. Inhibition of mycelial growth among all the strains after 3 days incubation on PDA amended with each compound, as indicated in the figure. Bars denote standard errors from three experiments. [file Image3.TIF]
