# Supplementary material for: Involvement of BcYak1 in the Regulation of Vegetative Differentiation and Adaptation to Oxidative Stress of Botrytis cinerea
Source: Front Microbiol. 2018 Feb 21;9:281. doi: 10.3389/fmicb.2018.00281 (PMC5826331; doi:10.3389/fmicb.2018.00281)
Supplement: Table S1 — Primers used in the study. [file Table1.docx]

Table S1

Primers used in the study

| Primer | Sequence(5'-3') | Relevant characteristics |
| --- | --- | --- |
| BcYak1-up-F | ATCCATCCATTCATCCATTCATTCA | PCR primers to amplify *BcYAK1* upstream fragment for the construction of *BcYak1* deletion mutants |
| BcYak1-up-R | GACCTCCACTAGCTCCAGCCAAGCCTTGGGGATGGGCTGGTGGGGGGTTT |  |
| BcYak1-down-F | ATAGAGTAGATGCCGACCGCGGGTTGCTTCACGATCGTAAATTTGTAGGA | PCR primers to amplify *BcYAK1* downstream fragment for the construction of *BcYak1* deletion mutants |
| BcYak1-down-R | TCATCATTAGACCTGTAGACACACC |  |
| BcYak1-out-F | ATCTCCACAGTATCGCCAAA | PCR primers for identification of *BcYAK1* deletion transformants |
| BcYak1-out-R | TGTCGCTGGTCTTCCATCATT |  |
| BcYak1-com-F | ATTgcggccgcCGTTGGTGTGCTTGACACATT | PCR primers to amplify full *BcYAK1* including 1148 bp up and 191 bp dowm fragment for complement of the *BcYAK1* deletion mutant |
| BcYak1-com-R | CGCgagctcCGGTTGGGTATCATGATTCA |  |
| BcYak1-R-F | AACCCCAGCTTCAGATACGAATCCTCG | PCR primers to introduce the K252R to *BcYAK1* |
| BcYak1-R-R | CGAGGATTCGTATCTGAAGCTGGGGTT |  |
| BcYak1-Q-F | AACCCCAGCTTCCAGTACGAATCCTCG | PCR primers to introduce the K252Q to *BcYAK1* |
| BcYak1-Q-R | CGAGGATTCGTACTGGAAGCTGGGGTT |  |
| HPH-F | GGCTTGGCTGGAGCTAGTGGAGGTC | PCR primers for amplification of the hygromycin resistant gene *HPH* |
| HPH-R | AACCCGCGGTCGGCATCTACTCTAT |  |
| β-tubulin-F | ACCGTTCCAGAGTTGACTCAA | PCR primers to amplify β-tubulin downstream fragment for the expression levels analysis |
| β-tubulin-R | GCAAGAAAGCCTTTCTTCTGA |  |
| BcTRR1-EX-F | ATGATCCAGCTACCGCATTGT | PCR primers to amplify *BcTRR1* downstream fragment for the expression levels analysis |
| BcTRR1-EX-R | AGGAATTTCTCGGCCTCAAGA |  |
| BcCCP1-EX-F | ACCGAGACGGAATTGATGAT | PCR primers to amplify *BcCCP1* downstream fragment for the expression levels analysis |
| BcCCP1-EX-R | TCTTCGAATACCCAACTCGA |  |

The respective exogenous enzyme sites are lowercase in the sequence.
